# Does ADHD Symptomatology Influence Treatment Outcome and Dropout Risk in Eating Disorders? A longitudinal Study

**DOI:** 10.3390/jcm9072305

**Published:** 2020-07-20

**Authors:** Giulia Testa, Isabel Baenas, Cristina Vintró-Alcaraz, Roser Granero, Zaida Agüera, Isabel Sánchez, Nadine Riesco, Susana Jiménez-Murcia, Fernando Fernández-Aranda

**Affiliations:** 1 Ciber Physiopathology, Obesity and Nutrition (CIBERObn), Instituto de Salud Carlos III, 08907 Barcelona, Spain; gtesta@idibell.cat (G.T.); ibaenas@bellvitgehospital.cat (I.B.); cvintro@bellvitgehospital.cat (C.V.-A.); Roser.Granero@uab.cat (R.G.); zaguera@bellvitgehospital.cat (Z.A.); isasanchez@bellvitgehospital.cat (I.S.); nriesco@bellvitgehospital.cat (N.R.); sjimenez@bellvitgehospital.cat (S.J.-M.); 2 Department of Psychiatry, Bellvitge University Hospital-IDIBELL, 08907 Barcelona, Spain; 3 Department of Psychobiology and Methodology of Health Sciences, Universitat Autònoma de Barcelona, 08193 Barcelona, Spain; 4 Department of Public Health, Mental Health and Maternal-Child Nursing, School of Nursing, University of Barcelona, 08907 Barcelona, Spain; 5 Department of Clinical Sciences, School of Medicine, University of Barcelona, 08907 Barcelona, Spain

**Keywords:** attention-deficit/hyperactivity disorder, ADHD, eating disorders, longitudinal, treatment outcome, dropout

## Abstract

Attention-deficit/hyperactivity disorder (ADHD) and its symptoms have been shown to be present in patients with eating disorders (EDs) and are associated with increased psychopathology and more dysfunctional personality traits. This study aimed to assess if the presence of ADHD symptoms in patients with EDs affects their short and long-term therapy outcome. A total of 136 consecutively treated ED patients were considered in this study. Baseline pre-treatment evaluation included the Adult ADHD Self-Report Scale (ASRS v1.1) for ADHD symptoms and the assessment of eating symptomatology using the Eating Disorders Inventory (EDI-2). Treatment outcome was evaluated in terms of ED symptoms after cognitive behavioral therapy (CBT) and dropout rate during treatment. Furthermore, we evaluated ED symptoms in treatment completers after a follow-up of 8 years on average. Path analyses assessed the potential mediational role of the EDI-2 total score in the relationship between ADHD and treatment outcome. Results showed that baseline symptoms of ADHD indirectly affected treatment outcome after CBT; the ASRS positive screening was related to higher eating symptomatology (standardized coefficient B = 0.41, *p* = 0.001, 95% CI: 0.26 to 0.55), and the presence of high ED levels contributed to the increase of dropout (B = 0.15, *p* = 0.041, 95% CI: 0.03 to 0.33) and a worse treatment outcome (B = 0.18, *p* = 0.041, 95% CI: 0.01 to 0.35). No direct effect was found between the ASRS positive screening with the risk of dropout (B = −0.08, *p* = 0.375) and worse treatment outcome (B = −0.07, *p* = 0.414). These results suggest the relevance of identifying specific treatment approaches for patients with ADHD symptoms and severe eating symptomatology.

## 1. Introduction

Attention-deficit/hyperactivity disorder (ADHD), which is characterized by symptoms of impulsivity, hyperactivity and inattention, has been described in relation to several psychiatric disorders including eating disorders (EDs) [1,2,3,4]. Accordingly, a higher prevalence of ADHD symptoms in EDs has been reported in many studies, in comparison with the general population [4,5,6,7,8,9,10]. Likewise, ADHD has been related with increased risk of disordered eating in childhood and adulthood [11,12,13,14], obesity [15] and behavioral addictions [16,17].

From a neurobiological perspective, a recent study examined the genetic factors common to both disorders and observed a stronger genetic association between ADHD and binge-eating behaviors [18]. From a clinical view, ADHD symptoms in ED were associated with higher ED symptomatology [19], specifically binge ED subtypes, with greater psychopathology [19,20,21], and increased levels of motor and cognitive impulsivity [22,23], as well as impulsive personality traits [24,25].

Despite the evidence linking ADHD and EDs, there is a lack of studies investigating whether ADHD symptoms may impact treatment response in patients with EDs, both in terms of treatment adherence (i.e., dropout rate) and outcome. To the best of our knowledge, there is only one previous study in female patients with EDs reporting higher ADHD symptoms at baseline as a predictor of non-recovery from eating-related symptomatology one year after treatment, especially for patients with loss of control overeating, bingeing and purging [26]. However, a high dropout rate (61%) was shown in that study, highlighting the need for future studies examining the impact of ADHD symptoms over dropout rate in EDs treatment. 

The first aim of the present study was to evaluate if the initial presence of ADHD symptomatology impacts the therapy outcome after a cognitive behavioral therapy (CBT) treatment for EDs and dropout rate during treatment. The second aim was to analyze whether the baseline ADHD symptomatology had an impact on long-term therapy outcome (after eight years on average). Furthermore, we assessed the influence of EDs severity over the relationship between ADHD symptoms and treatment results. 

## 2. Materials and Methods

### 2.1. Participants

**Figure 1:**
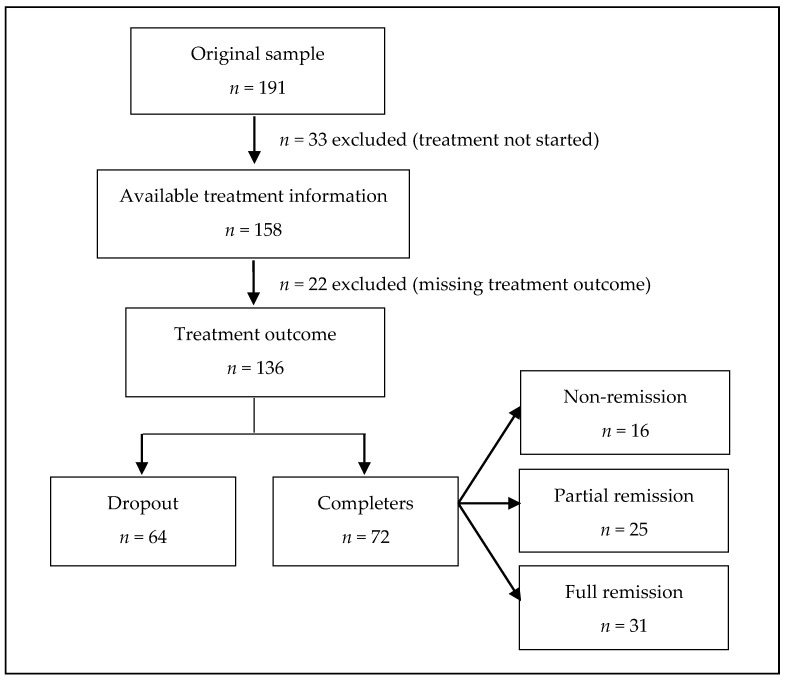
Flow chart for the sampling procedure.

The initial sample was comprised of 191 adult women diagnosed with an ED and presented for treatment to the Eating Disorder Unit within the Department of Psychiatry at Bellvitge University Hospital (HUB) (Barcelona, Spain) from 16 April 2009 to 31 January 2011. The baseline data of the initial sample was previously reported by Fernández-Aranda et al. (2013). Patients were diagnosed according to the DSM-IV-TR criteria [27], and diagnoses were reanalyzed and recodified post hoc using the DSM-5 criteria [28]. In the present study, we analyzed this sample of patients following a CBT treatment and after a longer follow-up (mean = 8.81 years; SD = 1.5). To that end, clinical records and an online shared medical network were analyzed retrospectively throughout the region of Catalonia (Spain). Thirty-three patients who did not start treatment were excluded from the original sample (*n* = 191); however, neither online nor paper clinical records were available for 22 out of 158 patients who started treatment. Thus, a final sample of 136 patients was included in the principal analysis. There were no baseline differences between patients who were included in this study and those who were not. Sixty-four participants included in the final sample did not complete treatment sessions (dropout) whereas 72 patients out of 136 completed the treatment (see flowchart; Figure 1). 

### 2.2. Assessment

Adult ADHD Self-Report Scale (ASRS-v1.1) [29]: The ASRS-v1.1 is a self-administered scale designed to screen ADHD in the adult population (aged 18 years and older). It comprises 6 items that are consistent with the DSM-IV-TR [27] criteria and address the manifestation of ADHD in adults; the first 4 questions examine the inattention component of ADHD, and the final 2, the hyperactivity component. Each item is assessed using a 5-point Likert scale (0–4). The total score ranges between 0 and 24, with the cut-off being set at 12. The Spanish validation reported good psychometric properties [30]. Internal consistency in the sample analyzed in this work was good (Cronbach’s alpha α = 0.77).

Eating Disorders Inventory-2 (EDI-2) [31]: This self-report questionnaire consists of 91 items, answered on a 6-point Likert scale, that assess different cognitive and behavioral characteristics related to ED. The EDI-2 provides standardized subscale scores as well as a global measure of ED severity, which can be obtained based on the sum of all the items. A Spanish version of this questionnaire [32] has been validated and it obtained excellent psychometrical properties as an external global measure of ED severity. Internal consistency for the total score in this study was good (Cronbach’s alpha α = 0.85).

### 2.3. Treatment

As described elsewhere [33,34], all patients received cognitive behavioral therapy (CBT) at HUB, which was carried out by clinical psychology experts in the field. The CBT group therapy intervention for bulimia nervosa (BN), binge eating disorder (BED) and other specified feeding or eating disorders (OSFED) consisted of 16 weekly outpatient sessions lasting 90 minutes each and a follow-up period of about two years’ duration that was carried out in a group format. Patients with BN first received six sessions of psychoeducational brief group therapy to offer information and psychoeducation about the negative consequences of BN. Patients diagnosed with anorexia nervosa (AN) completed a day hospital treatment program, which included two daily group CBT sessions during an average of 3 months. If they were severely underweight (IMC < 15 kg/m^2^), they were offered an in-patient treatment with a maximum duration of three months. Patients with AN also had a two-year follow-up period with individual/group sessions. The goals of the treatment were to train patients to implement CBT strategies to reduce eating symptoms and to enable patients to acquire good healthy habits. 

Patients were re-evaluated at discharge and categorized into the following three categories: full remission, partial remission and non-remission. According to DSM-5 criteria [28], the definition of full remission is a total absence of symptoms meeting diagnostic criteria for at least 4 consecutive weeks; partial remission is defined as a substantial symptomatic improvement but with residual symptoms; and patients who present poor outcomes are defined as non-remission. These categories were previously used to assess treatment outcome in threshold ED in other published studies [33,34]. Voluntary treatment discontinuation was categorized as dropout (i.e., not attending treatment for at least three consecutive sessions).

### 2.4. Follow-Up

A longitudinal follow-up of the patients was collected during an average period of 8.8 years (SD = 1.5), from the end of the first contact made in our unit to April 2020. This first contact coincided with the assessment using the ASRS screening questionnaire. By searching through centralized Catalonian clinical records and an online shared medical network, we assessed clinical evolution of all ED patients over the time, in terms of symptomatological remission (categorized as positive follow-up) or clinical non-remission of eating symptomatology (categorized as negative follow-up). From the total sample, clinical records were available for 123 patients, while 13 patients were missing (10.5% attrition rate). 

### 2.5. Ethics

The present study was carried out in accordance with the latest version of the Declaration of Helsinki. The Bellvitge University Hospital Clinical Research Ethics Committee approved the study and signed informed consent was previously obtained from all participants.

### 2.6. Statistical Analyses

Stata16 for Windows was used to carry out statistical analyses [35]. A comparison between the groups defined by the risk of dropout during the CBT (yes/no) and the classification of the therapy outcome (full/partial remission versus dropout/non-remission) was done with a chi-square test (*χ*^2^).

Survival function (Kaplan–Meier product-limit estimator) modeled the rate of dropout during the therapy. This procedure is usually used to describe the probability of the patients “living/surviving” for a certain amount of time after one concrete intervention [36]. In this study, “surviving” was defined for patients without dropout after the beginning of the CBT. 

Path analyses assessed the potential mediational role of the ED severity level (EDI-2 total score) in the relationship between ADHD and treatment outcome (i.e., therapy outcome after a CBT treatment and dropout rate during treatment). This procedure was defined in this work as a case of structural equation modeling (SEM), with the maximum-likelihood estimation method of parameter estimation, adjusting by the covariates participants’ age and the duration of the ED. The goodness-of-fit was evaluated using a standard statistical test, and it was considered adequate fitting for [37]: non-significant result (*p* > 0.05) in the *χ*^2^ test, root mean square error of approximation (RMSEA) < 0.08, Bentler’s comparative fit index (CFI) > 0.90, Tucker–Lewis Index (TLI) > 0.90 and standardized root mean square residual (SRMR) < 0.10. In this study, the relatively low sample size and the high correlations between the EDI-2 scores did not allow the SEM to be included in all the EDI-2 subscales registered for the participants (goodness-of-fit was not achieved). 

## 3. Results

### 3.1. Characteristics of the Sample

No statistical differences between participants selected for the study and those excluded were found for marital status (*χ*^2^ = 0.07, *p* = 0.982), education level (*χ*^2^ = 1.03, *p* = 0.317), socioeconomic status (*χ*^2^ = 2.67, *p* = 0.446), age (*T* = 1.04, *p* = 0.300), duration of the eating problems (*T* = 0.62, *p* = 0.535), onset of the ED (*T* = 0.70, *p* = 0.487), ED-symptom levels (*T* = 0.52, *p* = 0.603) and ADHD-symptom levels (*T* = 0.74, *p* = 0.462).

Table 1 shows the descriptive for the sociodemographic and clinical variables of the final sample included in the study. All participants were women, with a mean age of 28.7 years (SD = 9.6), most were single (66.9%) and had achieved a secondary education level (61.0%). The number of patients within the ASRS positive screening group was *n* = 46 (point prevalence estimate equal to 33.8%). Regarding CBT outcomes, the dropout rate in the total sample was 47.0%, non-remission was registered for 11.8%, partial remission for 18.4% and full remission for 22.8%. Within the completers subsample (*n* = 72), non-remission was registered for 22.2% of the participants, partial remission for 34.7% and full remission for 43.1%.

Table 2 includes the correlation matrix (Pearson correlation coefficients, R) between the ADHD level (measured through the ASRS scales) with the ED symptom level (EDI-2 scales). Due the strong relationship between the significance test for the correlation model and the sample size (high coefficients estimated in low samples sizes tend to show non-significance, while low coefficients tend to show significance obtained in samples with a large number of participants), Table 2 shows in bold the coefficients with an effect size that is in the moderate-mild (|R| > 0.24) to high-large range (|R| > 0.37). While moderate to high correlations were found between most EDI-2 scales, low correlation was found between ASRS inattention with hyperactive scores (R = 0.166). ASRS inattention positively correlated with all the EDI-2 scales, except for maturity fears, perfectionism and impulse regulation. ASRS hyperactivity also positively correlated with drive for thinness, body dissatisfaction, interoceptive awareness, impulse regulation and total. ASRS total correlated with all the EDI-2 scales (except for perfectionism), achieving the highest R-coefficient with the EDI-2 total (R = 0.497).

The ADHD level was not directly related to the short-term therapy outcomes in the study; the prevalence of the ASRS positive screening score was statistically equal for completers and dropouts, as well as for participants with partial remission and full remission versus those with dropout and non-remission (Table 3). No association was found even when stratified by the diagnostic subtype (Supplementary Table S1). Regarding data registered after the follow-up, neither was there any statistical association found between the ASRS positive screening with therapy outcome. 

**Figure 2:**
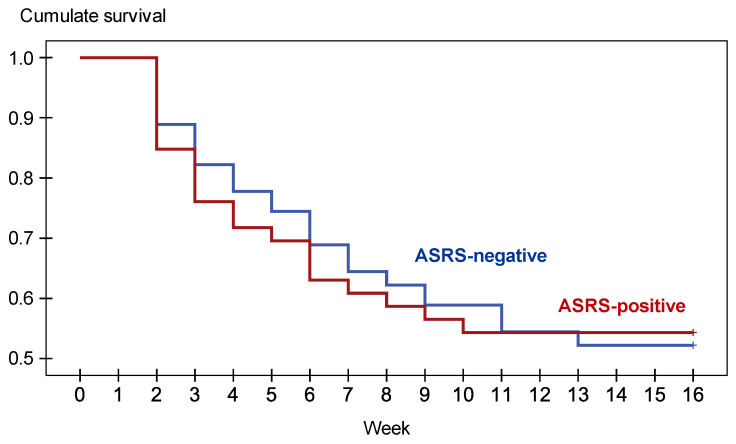
Survival function for dropout (*n* = 136).

Figure 2 includes the cumulate survival function for the time to dropout. The higher proportion of dropouts was registered during the two weeks after the beginning of the treatment (12.1% for the total sample), followed by the next two weeks (at the end of the first month 24.1% had followed). No statistical contribution was found between ASRS score with the rate to dropout (log rank test = 0.01, *p* = 0.995).

Supplementary Table S2 includes the associations between the ADHD levels (registered in the ASRS inattention, hyperactivity and total score) and the ED symptom levels (registered in the EDI-2 scores) with the treatment outcomes (during therapy, at short-term and in the follow-up). No statistical differences were found comparing the mean scores in the ASRS and the EDI-2 between the groups of the study.

### 3.2. Path Analysis

**Figure 3:**
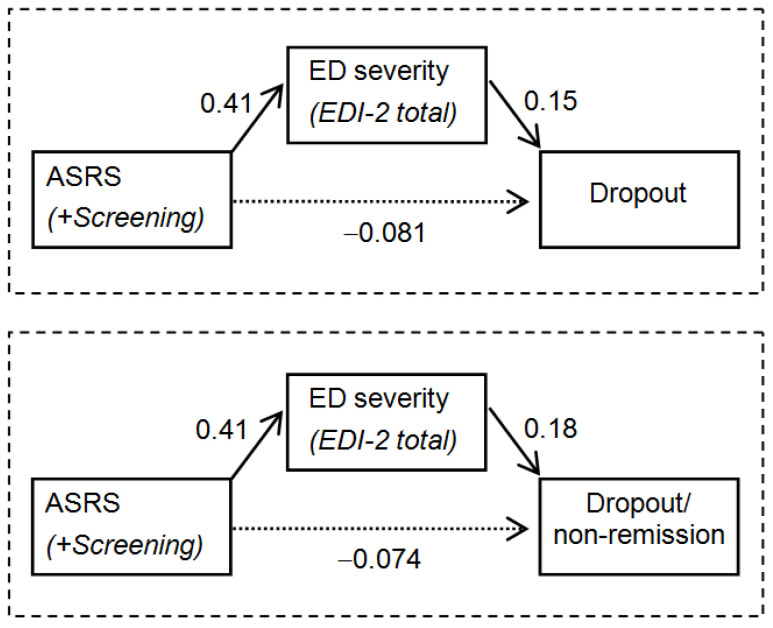
Path-diagrams: standardized coefficients in the SEM for the short-term therapy outcome (*n* = 136). Continuous line: significant coefficient. Dash line: non-significant coefficient. Results adjusted by age and duration of the ED.

Figure 3 shows the results of the SEM assessing the mediational link of the ED severity into the relationship between ADHD and the CBT outcomes. Goodness of fit was achieved for both models (risk of dropout: *χ*^2^ = 0.807 (*p* = 0.848), RMSEA = 0.001, CFI = 0.999, TLI = 0.998, SRMR = 0.015; better CBT outcome: *χ*^2^ = 0.029 (*p* = 0.864), RMSEA = 0.001, CFI = 0.999, TLI = 0.998, SRMR = 0.004). These results confirmed the indirect effect of ADHD on the therapy outcome; being in the ASRS positive screening group is related to higher ED severity (higher EDI-2 total), and this worse ED level contributed to the increase in the risk of dropout and a worse treatment outcome.

**Figure 4:**
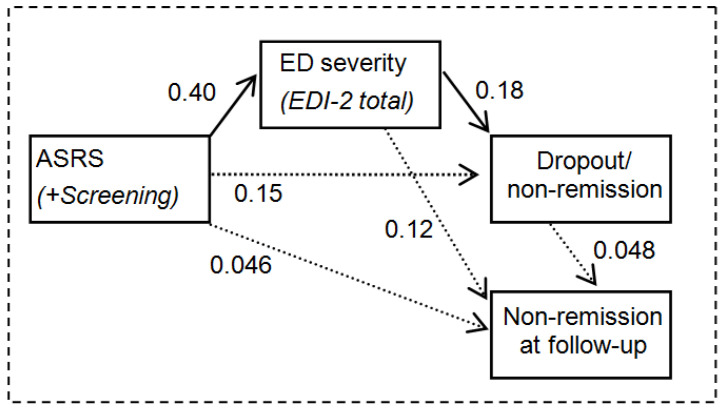
Path-diagrams: standardized coefficients in the SEM for the follow-up (*n* = 123). Continuous line: significant coefficient. Dash line: non-significant coefficient. Results adjusted by age and duration of the ED.

Figure 4 shows the results of the SEM adding the patients’ state at the end of the follow-up (adequate fitting was achieved: *χ*^2^ = 0.519 (*p* = 0.771), RMSEA = 0.001, CFI = 0.999, TLI = 0.998, SRMR = 0.016). This new model indicates that the ADHD screening score was not related (through a direct or indirect link) to the clinical condition measured in the follow-up.

## 4. Discussion

This study investigated whether the presence of ADHD symptoms in patients with EDs may impact treatment outcome and dropout for EDs. The second aim was to examine the influence of EDs severity on the relationship between ADHD symptoms and treatment outcome/dropout rate. Finally, a long-term follow-up was included to assess the effect of ADHD symptoms at baseline on the clinical evolution of EDs.

ADHD screening at baseline did not directly predict treatment outcome or dropout rate in patients attending a CBT for EDs. However, our results highlighted that the severity of eating symptomatology mediates the relation between ADHD and treatment outcome/dropout rate. Specifically, positive screening for ADHD symptoms was directly associated with the severity of EDI-2, which in turn is related to a higher dropout rate and a worse treatment outcome, in terms of dropout during treatment and non-remission of ED symptomatology.

The only previous study investigating the effect of ADHD over treatment for ED suggested that patients with higher ADHD symptoms presented a higher risk of poor post-treatment outcome, although the impact of ED severity on this relation was not assessed [26]. Interestingly, a high dropout from treatment was also reported in that study (61%), further suggesting the importance of taking into account dropout as a negative outcome of ED treatment. Considering that patients with ADHD tend to act impulsively, they may be more likely to abandon treatment, although limited information has been reported linking the effect of ADHD symptomatology in the treatment of comorbid psychopathology, in terms of dropout. These studies were mostly focused on substance use disorder (SUD), such as tobacco [38] or cocaine [39,40,41]. When SUD and ADHD are co-morbid, some factors seem to increase the risk of dropout, including: disruptive behaviors related to ADHD [40], cognitive deficit (e.g., executive dysfunctions) and the type of substance of abuse [41]. Concerning EDs, this is the first study suggesting that higher severity of EDs and co-occurring ADHD symptoms increase the risk of dropout.

In spite of the higher proportion of dropouts registered within the first two weeks of treatment, the ASRS score does not differ between early or later dropout, suggesting that ADHD symptoms may increase the dropout rate at different stages of treatment. Furthermore, we included a longer follow-up of the majority of the patients, showing that positive screening for ADHD symptoms at baseline did not affect the longer terms evolution of EDs. This is the first retrospective study which allows the evaluation of the impact of ADHD symptoms on EDs with a longitudinal follow-up of roughly 8 years. 

This study must be interpreted in light of its limitations. First, a self-reporting instrument was used to measure the frequency of adult ADHD symptoms, which may lead to an overestimation of this symptomatology. Therefore, future studies should focus on EDs patients with comorbidity with ADHD, not only taking into account ADHD symptomatology. Furthermore, comorbidity with others psychiatric disorders often associated with EDs (e.g., anxiety, depression) should be addressed by future studies in larger samples. Second, only female EDs patients were included in this study, so further studies would benefit from including male population as men report a higher prevalence of ADHD in adult life [42]. Third, it would have been advisable to have objective measures available after the follow-up. Finally, the retrospective nature of the design should be considered.

In conclusion, the present findings suggest a higher risk for dropout of treatment (i.e., CBT for EDs) and worse treatment outcomes in patients with more severe ED-symptoms, and an indirect effect of ADHD symptomatology on the treatment outcomes mediated by the ED severity levels. This was the first study to examine the effect of ADHD over the risk of dropout in patients treated for EDs, suggesting the relevance of identifying specific treatment approaches for patients with greater degrees of ADHD symptoms and severe EDs. 

## Figures and Tables

**Table 1:** Descriptive for the sample (*n* = 136).

Sociodemographic		*n*	%	Therapy Outcomes	*n*	%
Marital	Single	91	66.9%	Total sample (*n* = 136)		
	Married/Partner	34	25.0%	Dropout	64	47.0%
	Divorced/Separated	11	8.1%	Non-remission	16	11.8%
Education	Primary	32	23.5%	Partial remission	25	18.4%
	Secondary	83	61.0%	Full remission	31	22.8%
	University	21	15.5%	Completers subsample (*n* = 72)		
Age and ED severity level		Mean	SD	Non-remission	16	22.2%
Chronological age (years)		28.74	9.59	Partial remission	25	34.7%
EDI-2 total score		97.91	44.52	Full remission	31	43.1%
ADHD (ASRS screening)		*n*	*%*			
Positive screening		46	33.8%			
Negative screening		90	66.2%			

Note. SD: standard deviation.

**Table 2:** Correlation matrix for the Adult ADHD Self-Report Scale (ASRS) and Eating Disorders Inventory (EDI-2) scores (*n* = 136).

		2	3	4	5	6	7	8	9	10	11	12	13	14	15
1.	ASRS: inattention	0.166	**0.882 ^†^**	**0.252 ^†^**	**0.356 ^†^**	**0.384 ^†^**	**0.287 ^†^**	**0.287 ^†^**	**0.425 ^†^**	0.183	0.033	0.220	**0.312 ^†^**	**0.419 ^†^**	**0.420 ^†^**
2.	ASRS: hyperactive	---	**0.609 ^†^**	**0.372 ^†^**	**0.282 ^†^**	**0.341 ^†^**	0.226	0.086	0.207	0.214	0.116	**0.336 ^†^**	0.210	0.149	**0.342 ^†^**
3.	ASRS: total		---	**0.379 ^†^**	**0.419 ^†^**	**0.474 ^†^**	**0.337 ^†^**	**0.267**	**0.438 ^†^**	**0.246 ^†^**	0.078	**0.337 ^†^**	**0.346 ^†^**	**0.402 ^†^**	**0.497 ^†^**
4.	EDI-2: Drive.thinness			---	**0.622 ^†^**	**0.582 ^†^**	**0.367 ^†^**	**0.341**	**0.483 ^†^**	**0.297 ^†^**	**0.339 ^†^**	**0.364 ^†^**	**0.628 ^†^**	**0.440 ^†^**	**0.720 ^†^**
5.	EDI-2: Body.dissatisf.				---	**0.503 ^†^**	**0.483 ^†^**	**0.383**	**0.596 ^†^**	**0.283 ^†^**	0.211	**0.429 ^†^**	**0.506 ^†^**	**0.457 ^†^**	**0.751 ^†^**
6.	EDI-2: Interoc.awar.					---	**0.508 ^†^**	**0.500**	**0.700 ^†^**	**0.371 ^†^**	**0.305 ^†^**	**0.604 ^†^**	**0.666 ^†^**	**0.637 ^†^**	**0.838 ^†^**
7.	EDI-2: Bulimia						---	0.238	**0.430 ^†^**	0.032	0.056	**0.385 ^†^**	**0.486 ^†^**	**0.262 ^†^**	**0.562 ^†^**
8.	EDI-2: Interp.distrust							---	**0.561 ^†^**	**0.306 ^†^**	0.168	**0.347 ^†^**	**0.385 ^†^**	**0.697 ^†^**	**0.626 ^†^**
9.	EDI-2: Ineffectiveness								---	**0.503 ^†^**	**0.296 ^†^**	**0.568 ^†^**	**0.650 ^†^**	**0.749 ^†^**	**0.865 ^†^**
10.	EDI-2: Maturity fears									---	**0.277 ^†^**	**0.366 ^†^**	**0.279 ^†^**	**0.496 ^†^**	**0.549 ^†^**
11.	EDI-2: Perfectionism										---	**0.331 ^†^**	**0.314 ^†^**	**0.299 ^†^**	**0.438 ^†^**
12.	EDI-2: Impulse.regul.											---	**0.541 ^†^**	**0.576 ^†^**	**0.717 ^†^**
13.	EDI-2: Ascetic												---	**0.503 ^†^**	**0.768 ^†^**
14.	EDI-2: Social.insec.													---	**0.790 ^†^**
15.	EDI-2: Total score														---

Note. **^†^** Correlation with effect size in the moderate-mild (|R| > 0.24) to high-large range (|R| > 0.37).

**Table 3:** Description of ADHD measures within the groups defined by the short term therapy outcomes (*n* = 136) and the follow-up (*n* = 123).

Treatment Outcome	Completers (*n* = 72)		Dropout (*n* = 64)		*p*
**ADHD screening (+); *n*-%**	25	34.7%	21	32.8%	0.814
**Short-term therapy outcome**	Full/partial remission (*n* = 56)		Dropout/non-remission (*n* = 80)		*p*
ADHD screening (+); *n*-%	21	37.5%	25	31.3%	0.448
**Outcome at follow-up**	Remission (*n* = 87)		Non-remission (*n* = 36)		*p*
ADHD screening (+); *n*-%	29	33.3%	12	33.3%	1.00

## Supplementary Materials

The following are available online at https://www.mdpi.com/2077-0383/9/7/2305/s1, Table S1: Description of ADHD measures within the groups defined by the short-term therapy outcome (*n* = 136), Table S2: Description of ADHD measures within the groups defined by the short term therapy outcomes (*n* = 136) and the follow-up (*n* = 123).
