# Supplementary material for: Does ADHD Symptomatology Influence Treatment Outcome and Dropout Risk in Eating Disorders? A longitudinal Study
Source: J Clin Med. 2020 Jul 20;9(7):2305. doi: 10.3390/jcm9072305 (PMC7408799; doi:10.3390/jcm9072305)
Supplement: Supplementary file 1 [file jcm-09-02305-s001.pdf]

Table S1. Description of ADHD measures within the groups defined by the short-term therapy outcome (n=136).

	ADHD screening (+); n - %				
Risk for dropout	*Completers* *(n=72)*		*Dropout* *(n=64)*		*p*
Anorexia *(n=31)*	1	5.0%	2	18.2%	.235
Bulimia *(n=59)*	17	51.5%	12	46.2%	.683
OSFED *(n=26)*	4	50.0%	5	27.8%	.272
BED *(n=20)*	3	27.3%	2	22.2%	.795
Short therm therapy outcome	*Full/partial remission* *(n=56)*		*Dropout/non-remission* *(n=80)*		*p*
Anorexia *(n=31)*	1	6.7%	2	12.5%	.583
Bulimia *(n=59)*	15	57.7%	14	42.4%	.244
OSFED *(n=26)*	2	50.0%	7	31.8%	.482
BED *(n=20)*	3	27.3%	2	22.2%	.795

Note. OSFED: other specified feeding eating disorder. BED: binge eating disorder

Table S2. Description of ADHD measures within the groups defined by the short term therapy outcomes (n=136) and the follow-up (n=123).

	Treatment outcome					Short-term therapy outcome					Outcome at follow-up				
	Completers treatment n=72		Dropout treatment n=64			Full/partial remission n=56		Dropout/non-remission n=80			Remission n=87		Non-remission n=36		
	Mean	SD	Mean	SD	p	Mean	SD	Mean	SD	p	Mean	SD	Mean	SD	p
ASRS: inattention	6.85	3.57	7.06	2.73	.696	7.21	3.46	6.76	3.00	.419	7.07	3.36	6.42	3.08	.318
ASRS: hyperactive	4.49	2.08	4.80	1.63	.337	4.54	2.12	4.70	1.69	.617	4.55	1.90	4.97	1.83	.262
ASRS: total	11.33	4.40	11.89	3.44	.416	11.75	4.19	11.49	3.84	.706	11.62	4.22	11.44	3.64	.827
EDI-2: Drive.thinness	13.88	6.14	14.30	5.69	.678	13.54	6.33	14.46	5.62	.376	13.67	5.80	14.81	6.10	.331
EDI-2: Body.dissatisf.	16.19	8.87	17.89	7.96	.248	16.36	9.07	17.43	8.04	.470	16.61	8.71	17.44	7.66	.618
EDI-2: Interoc.awar.	10.11	6.96	11.70	6.82	.185	10.25	6.99	11.28	6.88	.397	10.14	6.70	12.33	7.46	.112
EDI-2: Bulimia	6.21	4.99	7.10	5.37	.322	6.30	4.86	6.85	5.40	.549	6.67	5.31	6.89	4.94	.830
EDI-2: Interper.distrust	5.06	4.41	5.48	4.17	.571	5.20	4.60	5.29	4.07	.900	5.16	4.19	5.39	4.47	.788
EDI-2: Ineffectiveness	9.56	7.16	11.49	7.50	.127	9.75	7.25	10.96	7.43	.347	10.14	7.65	11.03	7.08	.550
EDI-2: Maturity fears	7.22	5.36	7.98	6.07	.440	7.43	5.55	7.68	5.82	.799	7.55	5.91	7.50	5.36	.964
EDI-2: Perfectionism	5.18	4.35	6.10	3.88	.203	4.64	4.23	6.29	3.98	.052	5.24	3.93	6.08	4.67	.309
EDI-2: Impulse.regulat.	5.17	5.54	6.87	6.00	.088	5.29	5.70	6.44	5.86	.255	5.17	5.37	7.42	6.57	.051
EDI-2: Ascetic	6.74	4.14	7.22	4.23	.501	6.64	4.19	7.19	4.17	.455	6.51	3.97	7.78	4.30	.117
EDI-2: Social insecurity	7.22	5.07	7.90	5.10	.438	7.32	5.24	7.70	4.99	.674	7.48	5.04	7.28	5.54	.842
EDI-2: Total score	92.53	45.03	103.97	43.49	.135	92.71	46.87	101.55	42.71	.256	94.33	44.96	103.94	44.95	.283

Note. SD=standard deviation.
